# 13-year mortality trends among hospitalized patients with inflammatory bowel disease

**DOI:** 10.1186/1471-230X-12-79

**Published:** 2012-06-26

**Authors:** Justin L Sewell, Hal F Yee

**Affiliations:** 1 Center for Innovation in Access and Quality, Department of Medicine, Division of Gastroenterology and Hepatology, San Francisco General Hospital, University of California San Francisco, San Francisco, CA, USA

**Keywords:** Inflammatory bowel disease, Crohn’s disease, Ulcerative colitis, Hospitalization, Epidemiology, Mortality, In-hospital mortality, Outcomes

## Abstract

**Background:**

Studies document increasing rates of hospitalization among patients with inflammatory bowel disease, but temporal trends for in-hospital mortality among patients with inflammatory bowel disease are not characterized. We sought to determine whether in-hospital mortality changed over a 13-year period among nationwide hospitalizations associated with inflammatory bowel disease. We additionally sought to identify factors correlated with mortality.

**Methods:**

We used the National Hospital Discharge Survey, a large nationally representative database, for the years 1994 through 2006. Age- and mortality-adjusted rates of in-hospital mortality and standardized mortality ratios were calculated for four time periods. Logistic regression analysis was used to assess associations between advancing time and mortality in adjusted analyses.

**Results:**

150 (0.9%) of 17,393 hospitalizations for patients with inflammatory bowel disease ended in death. Age-adjusted in-hospital mortality decreased from 3.6 deaths per 1,000 hospital days in 1994–96 to 2.4 per 1,000 in 2003–06; standardized mortality ratio decreased from 0.33 to 0.27. Similar trends were seen for patients with ulcerative colitis, but mortality did not change over time among patients with Crohn’s disease. Multivariable logistic regression analysis confirmed the significance of these changes in mortality, with 17% decreased odds of in-hospital death per three-year period (*P* = 0.012). Subject age (OR 1.06 per year, *P* < 0.001), Charlson comorbidity index (OR 1.29 per 1-point increase, *P* < 0.001), and diagnosis of ulcerative colitis (versus Crohn’s disease, OR 1.41, *P* = .042) were also associated with in-hospital mortality.

**Conclusions:**

The odds of in-hospital mortality among hospitalized patients with inflammatory bowel disease decreased by 17% per 3-year period from 1994 to 2006 in analysis adjusted for age and comorbidity status, in this large, nationally representative database. Multiple factors likely contribute to these trends.

## Background

The inflammatory bowel diseases (Crohn’s disease and ulcerative colitis) are chronic, lifelong diseases that affect nearly 1.5 million people in the United States and are associated with significant morbidity [1]. Although management of patients with inflammatory bowel disease (IBD) continues to evolve, measuring ambulatory IBD-related outcomes in the US is difficult due to the fragmented nature of the healthcare system. Patients with IBD are at increased risk for hospitalization, with inpatient care accounting for one-third of IBD-related healthcare costs [2]. In contrast with the ambulatory setting, nationally representative databases that record patient outcomes, including in-hospital mortality, are publicly available. In-hospital mortality is a clinically important outcome that may be affected by changes in IBD management over time. Although studies have reported point estimates of in-hospital mortality [3,4], temporal trends for in-hospital mortality among IBD patients are not documented.

To address this gap in knowledge, we sought to determine whether age- and comorbidity adjusted rates of in-hospital mortality changed among hospitalized IBD patients from 1994 to 2006, using a large, nationally representative sample of hospitalizations in the United States. We additionally sought to identify variables correlated with in-hospital mortality among patients with IBD.

## Methods

### Study design

We obtained data from the National Hospital Discharge Survey (NHDS) for years 1994 through 2006. Data from each survey year were downloaded electronically, where they are publicly available through the NHDS website. Hospitalizations including a diagnosis of Crohn’s disease or ulcerative colitis were identified using International Classification of Diseases, 9^th^ edition (ICD9) codes.

### Data source

The NHDS is an annual survey administered by the Centers for Disease Control [5,6]. It collects data from a national probability sample of approximately 500 hospitals in the United States, and includes approximately 300,000 hospitalizations annually. Federal and military hospitals, hospitals in the Department of Veterans Affairs, hospitals of institutions (such as prisons), and hospitals with five or fewer inpatient beds are excluded. Data collected include demographics, hospital characteristics, hospitalization data, up to seven ICD9 codes, and up to four procedure codes. Data collection and processing methods produce an unbiased, nationally representative sample of hospital discharges each year. No personally identifying data are included, so, the unit of measurement is by hospitalization, rather than by patient.

### Inclusion and exclusion criteria

Hospitalizations including ICD9 codes 555.xx (for Crohn’s disease) or 556.xx (for ulcerative colitis) were identified. Records including diagnostic codes for both Crohn’s disease and ulcerative colitis were categorized as having the disease that was listed first in order. We excluded children based on age of 17 years and younger. We further excluded patients over the age of 99, as patients age 100 and older are given an age of “202” in the NHDS, which would interfere with statistical analysis.

### Variables, data sources & measurement

The dependent variable and primary outcome was in-hospital mortality, which was identified by discharge condition of “dead”. We calculated mortality rates per 1,000 hospital days, which were directly age-adjusted and mortality-adjusted (using the Charlson comorbidity index) to the total NHDS population. We calculated the Charlson Index using ICD9 codes present within the NHDS. Independent variables obtained directly from the NHDS included age, gender, race, geographical region, and hospital size. We characterized the primary reason for hospitalization as the first listed ICD9 code.

### Statistical analysis

We performed univariable analyses to compare IBD-associated hospitalizations ending in death versus those not ending in death. We compared categorical variables using *χ*^2^ tests or Fisher’s exact test, as appropriate, and continuous variables using two-tailed t-tests.

We divided the 13 years studied into three 3-year time periods (1994–96, 1997–99, 2000–02) and one 4-year time period (2003–06) for statistical analysis. We calculated age- and comorbidity-adjusted in-hospital mortality rates, and standardized mortality ratios (SMRs), using the overall adult NHDS population as the reference standard. We used multivariable logistic regression analysis to evaluate the statistical significance of time period on odds of death in models adjusted for variables of clinical and statistical significance.

We performed three different sensitivity analyses. The first excluded hospitalizations less than 2 days in length. The second included only patients with a *primary* diagnosis of ulcerative colitis or Crohn’s disease (defined as the first ICD9 code listed in the NHDS). The third sensitivity analysis excluded hospitalizations with a primary diagnosis of a cardiopulmonary disease that was among the top 10 primary diagnoses in the overall sample.

Analyses were performed using Stata, versions 10 and 11 (Statacorp, College Station, TX). *P*-values of .05 or less were the standard for statistical significance.

### Ethical considerations

The Institutional Review Board of the University of California San Francisco granted exempt status to this study, because it is an analysis of publicly available data and includes no identifying information of any kind.

## Results

### Study population & univariable associations with mortality

17,393 hospitalizations included a diagnosis of IBD; 150 (0.9%) ended in death (Table 1). Subjects who died were older than subjects who did not die (70.3 years versus 49.2 years, *P* < 0.0001). Thirty-nine percent of hospitalizations ending in death included a primary diagnosis of IBD compared with 46% of hospitalizations not ending in death (P = NS). Hospitalizations with ulcerative colitis were twice as likely to end in death as hospitalizations with Crohn’s disease (1.3% versus 0.6%, *P* < 0.001). Hospitalizations ending in death were associated with significantly higher Charlson indices than those not ending in death (0.7 versus 2.0, P < .001).

**Table 1:** Characteristics of hospitalizations alive at discharge versus those dead at discharge

	**Alive at discharge *N* ** **= 17,243**	**Dead at discharge *N* ** **= 150**	** *P* -value**^**a**^
Age, years, mean (SD)	49.2 (18.8)	70.3 (16.1)	<0.0001
Gender, No. (%)			
Female	9,977 (57.9)	86 (57.3)	
Male	7,266 (42.1)	64 (42.7)	
Race, No. (%)			
White	10,504 (60.9)	104 (69.3)	
Black	1,569 (9.1)	7 (4.7)	0.03^b^
Asian	66 (0.4)	0 (0.0)	
Native American	54 (0.3)	0 (0.0)	
Other	371 (2.2)	1 (0.7)	
Unknown	4,679 (27.1)	38 (25.3)	
Geographical region, No. (%)			
Northeast	4,214 (24.4)	40 (26.7)	
Midwest	5,418 (31.4)	42 (28.0)	0.05^c^
South	5,637 (32.7)	43 (28.7)	0.04^c^
West	1,974 (11.5)	25 (16.7)	
Number of beds in hospital, No. (%)			
6-199 beds	5,482 (31.8)	56 (37.3)	
200-499 beds	9,250 (53.6)	73 (48.7)	
500+ beds	2,511 (14.6)	21 (14.0)	
Disease subtype, No. (%)			<0.001
Crohn’s disease	11,149 (64.7)	71 (47.3)	
Ulcerative colitis	6,094 (35.3)	79 (52.7)	
IBD as primary diagnosis, No. (%)^d^	7,929 (46.0)	59 (39.3)	
Charlson comorbidity index, mean (SD)	0.7 (1.3)	2.0 (1.4)	<0.001

^a^ For categorical variables with more than two responses, comparisons were made between all possible pairs; only significant *P*-values are reported.

^b^ Compared with whites.

^c^ Compared with West region.

^d^ A “primary” diagnosis of IBD indicates that the first-listed ICD9 code was either 555 or 556.

### Top diagnoses

Ulcerative colitis was the primary diagnosis in 2,989 (49.0%) of ulcerative colitis-associated hospitalizations. Crohn’s diseases was the primary diagnosis in 4,999 (44.8%) of Crohn’s disease-associated hospitalizations. Fifty-nine (0.74%) hospitalizations with IBD as the primary diagnosis ended in death, as compared with 91 (0.97%) of hospitalizations with IBD as a non-primary diagnosis (*P* = NS).

Table 2 summarizes the top 10 primary diagnoses for IBD-associated hospitalizations ending in death and not ending in death. Five diagnoses were among the top 10 in both groups: Crohn’s disease, ulcerative colitis, other disorders of the intestine, pneumonia, and other forms of chronic ischemic heart disease. Crohn’s disease and ulcerative colitis were statistically more common primary diagnoses among hospitalizations not ending in death. Five primary diagnoses related to cardiac and pulmonary disease were statistically more common in hospitalizations ending in death.

**Table 2:** Top 10 primary diagnoses among inflammatory bowel disease-associated hospitalizations, by death status

**ICD-9 code**^a^	**Alive at discharge, No. (%)**^**b**^	**Dead at discharge, No. (%)**^**b**^
555 (Crohn’s disease)^c,d^	**4977 (28.9)**	**22 (14.7)**
556 (ulcerative colitis)^c,d^	**2952 (17.1)**	**37 (24.7)**
560 (intestinal obstruction without mention of hernia)	**737 (4.3)**	2 (1.3)
276 (disorders of fluid, electrolyte, and acid–base balance)	**323 (1.9)**	1 (0.7)
569 (other disorders of intestine)^c^	**322 (1.9)**	**4 (2.7)**
296 (episodic mood disorders)	**228 (1.3)**	1 (0.7)
577 (diseases of pancreas)	**211 (1.2)**	0 (0.0)
486 (pneumonia)^c^	**206 (1.2)**	**4 (2.7)**
414 (other forms of chronic ischemic heart disease)^c,e^	**195 (1.1)**	**3 (2.0)**
578 (gastrointestinal hemorrhage)	**179 (1.0)**	1 (0.7)
410 (acute myocardial infarction)^d,e^	105 (0.6)	**6 (4.0)**
162 (malignant neoplasm of trachea, bronchus and lung)^d^	13 (0.007)	**5 (3.3)**
491 (chronic bronchitis)^d,e^	134 (0.8)	**5 (3.3)**
427 (cardiac dysrhythmias)^d,e^	153 (0.9)	**4 (2.7)**
428 (heart failure)^d,e^	105 (0.6)	**3 (2.0)**

^a^ Diagnoses in the left column include those that were among the 10 most common primary diagnoses for patients who were alive and dead at the time of discharge. Order of variable listing reflects the relative frequency among patients who were alive at discharge, followed by patients who were dead at discharge.

^b^ Values in bold indicate that the diagnosis was among the top 10 for that column.

^c^ Diagnosis is among the top 10 primary diagnoses for those alive at discharge *and* those dead at discharge.

^d^ P < 0.05 comparing proportions among hospitalizations ending in death versus those not ending in death.

^e^ ICD9 code used to exclude hospitalizations from third sensitivity analysis.

### In-hospital mortality: IBD

**Figure 1:**
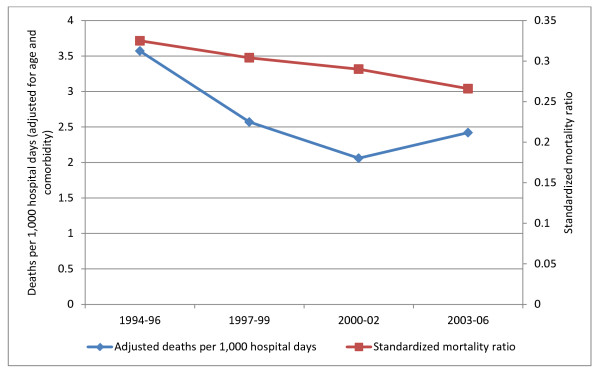
Mortality trends over time for hospitalizations associated with IBD.

For hospitalizations associated with either ulcerative colitis or Crohn’s disease, age- and comorbidity-adjusted rates of in-hospital mortality decreased from 3.6 deaths per 1,000 hospital days in 1994–96 to 2.1 deaths per 1,000 hospital days in 2000–02; there was subsequent slight increase to 2.4 deaths per 1,000 hospital days in 2003–06 (Figure 1). The standardized mortality ratio decreased from 0.33 in 1994–96 to 0.27 in 2003–06 (Figure 1). In multivariable logistic regression analysis, the odds of mortality among all hospitalizations with IBD decreased by 17% per time period studied year (*P* = 0.012, Table 3). Hospitalizations with ulcerative colitis had 41% increased odds of mortality compared with Crohn’s disease-related hospitalizations (*P* = 0.042). Age and Charlson index were both positively associated with mortality (*P* < 0.001 for both comparisons).

**Table 3:** Multivariable logistic regression of factors associated with mortality

	**OR (95% CI)**	** *P* -value**
**All patients with IBD**		
Calendar period^a^	0.83 (0.72,0.96)	0.012
Age, per year	1.06 (1.05,1.07)	<0.001
Charlson index	1.29 (1.21,1.38)	<0.001
Diagnosis of ulcerative colitis (versus Crohn’s disease)	1.41 (1.01,1.95)	0.042
**Patients with ulcerative colitis**		
Calendar period^a^	0.80 (0.66,0.97)	0.02
Age, per year	1.06 (1.04,1.08)	<0.001
Charlson index	1.27 (1.15,1.40)	<0.001
**Patients with Crohn’s disease**		
Age, per year	1.06 (1.04,1.07)	<0.001
Charlson index	1.31 (1.19,1.45)	<0.001

^a^ Compares four calendar periods studied; odds ratio represents odds per advancing calendar period.

### In-hospital mortality: Ulcerative colitis

**Figure 2:**
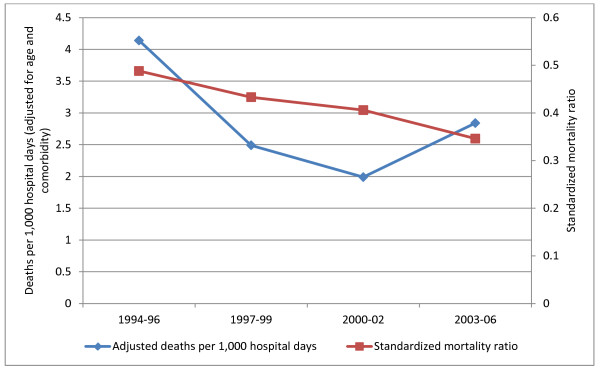
Mortality trends over time for hospitalizations associated with ulcerative colitis.

For hospitalizations associated with ulcerative colitis, the age- and comorbidity-adjusted rate of in-hospital mortality decreased from 4.1 deaths per 1,000 hospital days in 1994–96 to 2.0 deaths per 1,000 hospital days in 2000–02; there was subsequent increase to 2.8 deaths per 1,000 hospital days in 2003–06 (Figure 2). The standardized mortality ratio decreased from 0.49 in 1994–96 to 0.35 in 2003–06 (Figure 2). In multivariable logistic regression analysis, odds of mortality decreased by 20% per time period studied (*P* = 0.02, Table 3). Age and Charlson index both retained a strong positive association with mortality (*P* < .001 for both variables).

### In-hospital mortality: Crohn’s disease

For hospitalizations associated with Crohn’s disease, the age- and comorbidity-adjusted rate of in-hospital mortality decreased numerically from 2.7 deaths per 1,000 hospital days in 1994–96 to 1.8 deaths per 1,000 hospital days in 2000–02, and then increased to 2.1 deaths per 1,000 hospital days in 2003–06. The standardized mortality ratio was unchanged over the study period (0.23 in 1994–96 and 1997–99, and 0.22 in 2000–02 and 2003–06). In multivariable logistic regression, calendar period was not significantly associated with mortality. However, age and Charlson index had significant positive associations with mortality (*P* < 0.001 for both variables, Table 3).

### Sensitivity analyses

In logistic regression analysis excluding hospitalizations of less than 2 days’ duration, there were only 20 deaths among IBD associated hospitalizations. Diagnosis of ulcerative colitis was no longer statistically associated with death. However, age, Charlson index, and time period remained significantly associated with in-hospital mortality, with magnitudes of association similar to the initial logistic regression analysis. Specifically, the odds of in-hospital mortality decreased by 15% per calendar period (adjusted OR 0.85, 95% confidence interval 0.73,0.99). In a similar sensitivity analysis of ulcerative colitis-associated hospitalizations, time period retained a significant statistical association (adjusted OR 0.80, 95% CI 0.65,0.99).

In logistic regression analysis limited to hospitalizations including either Crohn’s disease or ulcerative colitis as the *primary* diagnosis there were 59 deaths. Only Charlson index and age retained significant associations with in-hospital mortality with magnitude of association similar to the original logistic regression analysis. In this sensitivity analysis, time period no longer retained a significant association with in-hospital mortality (adjusted OR 1.11, 95% confidence interval 0.88,1.41).

In logistic regression analysis excluding hospitalizations with a primary diagnosis of a cardiopulmonary disease among the top 10 diagnoses (see Table 2), there were 129 deaths. Time period retained a significant association with death, with odds of mortality decreasing by 14% per advancing time period (OR 0.86, 95% CI 0.735,0.996). Age and Charlson index retained significant positive associations with death, with magnitude similar to the original regression analysis. However, ulcerative colitis was no longer associated with increased mortality risk.

## Discussion

The management of IBD continues to evolve rapidly, but temporal trends in population-level mortality have not been previously reported. In-hospital mortality is an important clinical outcome readily available through nationally representative data (as opposed to ambulatory outcomes, for which nationally representative data are not readily available). In this study, we found that, over the 13-year period from 1994 to 2006, the odds of in-hospital mortality among hospitalizations associated with IBD decreased by 17% per time period studied in analysis adjusted for age and comorbidity. These trends seem to be driven largely by hospitalizations associated with ulcerative colitis. Adjusted analyses suggest that trends are not likely explained by temporal changes in age or comorbidity.

Prior studies of in-hospital mortality for patients with IBD used samples collected through the Nationwide Inpatient Sample ranging from 1998 to 2005. These studies reported point estimates of in-hospital mortality, but did not examine temporal trends in in-hospital mortality [3,4,7-11]. Mortality rates for unselected IBD patients in these studies ranged from 0.3 to 1.3% of hospital admissions, or 0.6 to 1.2 deaths per 1,000 hospital days. We found similar mortality rates, supporting the accuracy of our primary outcome measure. Most patients who died in our study were age 60 years or older. This is consistent with prior studies, which showed that IBD confers a markedly increased mortality risk among patients age 60 years and older [12].

Although our data do not permit exact identification of driving forces behind these trends, they do permit some speculation. Our analyses were adjusted for age and comorbid status, using the Charlson index, which is known to be an accurate measure of comorbidities. The fact that time period remained significantly associated with mortality suggests that reductions in mortality are not primarily due to confounding by temporal trends in age or comorbidity among hospitalizations included in the NHDS. The NHDS uses a similar sampling protocol each year, so our results are not likely confounded by measurement bias. Furthermore, it is unlikely that the disease process of IBD itself changed dramatically over the 13 year time-period of our study.

The above factors lead to natural speculation that improvements in healthcare could contribute to reductions in in-hospital mortality. Our sensitivity analyses allow some commentary in three specific areas. The sensitivity analysis limited to hospitalizations with a primary diagnosis of IBD suggests that an improvement in IBD care alone is not likely to account for reductions in mortality. However, this analysis included a small number of deaths (59 deaths) spread out over a 13-year period, limiting the power of the analysis. Improvements in IBD care would probably be better assessed in an ambulatory setting. The sensitivity analysis excluding hospitalizations with a primary diagnosis of cardiopulmonary disease suggests that improved care for cardiopulmonary diseases is not the sole driver of reductions in mortality. Finally, the sensitivity analysis excluding brief hospitalizations suggests that trends are not likely due more frequent hospitalizations for less ill patients, who would likely have brief hospitalization durations. It is likely than numerous factors contribute to the mortality trends that we document. Importantly, though, our analyses argue against confounding by changing age, comorbidity, or hospitalization practices.

It is interesting that mortality trends among IBD-associated hospitalizations appeared to be driven primarily by hospitalizations associated specifically with ulcerative colitis. Although ulcerative colitis accounted for only a third of IBD-associated hospitalizations, more than half of deaths were in hospitalizations with ulcerative colitis, and ulcerative colitis conferred a 41% increased odds of death compared with Crohn’s disease. Furthermore, mortality reduced significantly over time in all IBD hospitalizations, and in ulcerative colitis hospitalizations specifically, but not in Crohn’s disease hospitalizations. Compared with Crohn’s disease, hospitalizations with ulcerative colitis were associated with a significantly higher mean age and Charlson index, but these did not vary over time in ulcerative colitis related hospitalizations (data not shown). Furthermore, the proportion of ulcerative colitis associated hospitalizations with a primary (versus secondary) diagnosis of IBD did not change over time (data not shown).

A major impetus for this study was the lack of studies documenting temporal trends in IBD-related outcomes. We chose to use inpatient data due to the lack of nationally representative ambulatory data for patients with IBD. Much of what we know about the course of IBD is drawn from cohorts that, while well-characterized, are not nationally representative [13-15]. This limits understanding of changes in IBD course over time at a population level, and underscores the need for prospectively collected, nationally representative, ambulatory data in IBD.

## Conclusions

In summary, we document decreasing in-hospital mortality among hospitalizations with IBD in the United States from 1994 to 2006 in this large, nationally representative database, using analyses adjusted for the important confounders of age and comorbidity. Multiple factors likely contribute to these trends.

## Abbreviations

CI: 95% confidence interval; IBD: Inflammatory bowel disease; ICD9: International classification of diseases, 9th edition; NHDS: National hospital discharge survey; OR: Odds ratio; SD: Standard deviation.

## Competing interests

The authors declare that they have no competing interests.

## Authors’ contributions

JS developed study question, downloaded data, cleaned database, performed statistical analysis, and wrote manuscript. HY provided supervised development of study question and design and revised the manuscript for intellectual content. Both authors approve of the final version for submission.

## Pre-publication history

The pre-publication history for this paper can be accessed here:

http://www.biomedcentral.com/1471-230X/12/79/prepub
